# Automated analysis of co-localized protein expression in histologic sections of prostate cancer

**DOI:** 10.1371/journal.pone.0178362

**Published:** 2017-05-26

**Authors:** Thomas A. Tennill, Mitchell E. Gross, Hermann B. Frieboes

**Affiliations:** 1 Department of Bioengineering, University of Louisville, Louisville, KY, United States of America; 2 Lawrence J. Elliston Institute for Transformational Medicine, University of Southern California, Los Angeles, CA, United States of America; 3 James Graham Brown Cancer Center, University of Louisville, Louisville, KY, United States of America; University of Oklahoma Health Sciences Center, UNITED STATES

## Abstract

An automated approach based on routinely-processed, whole-slide immunohistochemistry (IHC) was implemented to study co-localized protein expression in tissue samples. Expression of two markers was chosen to represent stromal (CD31) and epithelial (Ki-67) compartments in prostate cancer. IHC was performed on whole-slide sections representing low-, intermediate-, and high-grade disease from 15 patients. The automated workflow was developed using a training set of regions-of-interest in sequential tissue sections. Protein expression was studied on digital representations of IHC images across entire slides representing formalin-fixed paraffin embedded blocks. Using the training-set, the known association between Ki-67 and Gleason grade was confirmed. CD31 expression was more heterogeneous across samples and remained invariant with grade in this cohort. Interestingly, the Ki-67/CD31 ratio was significantly increased in high (Gleason ≥ 8) versus low/intermediate (Gleason ≤7) samples when assessed in the training-set and the whole-tissue block images. Further, the feasibility of the automated approach to process Tissue Microarray (TMA) samples in high throughput was evaluated. This work establishes an initial framework for automated analysis of co-localized protein expression and distribution in high-resolution digital microscopy images based on standard IHC techniques. Applied to a larger sample population, the approach may help to elucidate the biologic basis for the Gleason grade, which is the strongest, single factor distinguishing clinically aggressive from indolent prostate cancer.

## Introduction

Prostate cancer is the second most common form of cancer for men in the United States, with an incidence of over 180,000 and annual mortality of 26,000 deaths expected for 2016 [1]. From the time of diagnosis, treatment modalities vary widely and may include surgery (complete removal of the prostate), radiation (delivered to the prostate with or without adjacent lymph nodes), or conservative therapy (surveillance without any form of active therapy). Similarly, the prognosis for newly diagnosed patients varies widely. Some patients are diagnosed with low-risk disease, unlikely to progress or require treatment within the individual’s lifetime, while other patients are diagnosed with aggressive, high-risk disease which may entail significant morbidity and mortality in a relatively short time. It is critical to accurately assess a patient’s risk of morbidity or mortality during the individual’s expected lifetime. Standard features incorporated into prognostic algorithms include clinical T-stage and prostate specific antigen (PSA) level in addition to pathologic criteria. As most patients are diagnosed with clinically non-palpable (T1) disease with relatively low PSA values (generally < 10 ng/dl), pathologic features factor heavily in risk prognostication.

Histologic grade (Gleason grade) is the major pathologic factor clinically employed to determine risk prognostication. In typical practice, the Gleason grade is based on histologic patterns of neoplastic glandular growth; e.g., the growth of discrete glandular units in and amongst non-neoplastic acini is termed pattern 3, ill-defined glands with poorly formed lumina or cribiform structures are termed pattern 4, and a solid nest of cancer cells or single cells is termed pattern 5 [2]. The Gleason score is then derived by adding the patterns observed in different areas to produce a sum typically ranging from 6 to 10. The score is the single most important clinical variable in many risk prognostication systems [3–7] and has even been found to add independent information to prognostic nomograms for recurrent prostate cancer occurring long after an initial diagnostic biopsy [8].

Various studies have shown that microvessel density or epithelial proliferation may augment conventional Gleason grading. CD31 (a.k.a. platelet-endothelial cell adhesion molecule type 1) is an immunohistochemical stain that indicates presence of endothelial cells, granulocytes, monocytes, and platelets. Microvessel index, defined by an arrangement of CD31+ endothelial cells, has been associated with tumor aggressiveness and metastasis in patients with prostate cancer [9, 10]. Ki-67 is a nuclear non-histone protein that is present in all stages of the cell cycle except G0. As proliferating cells express Ki-67, it can be used to estimate the growth fraction of both benign and malignant tissue. In prostate cancer, Ki-67 expression has been correlated with Gleason grade and poor clinical outcomes [11]. Thus, while vascularity and proliferation have independently been shown to contribute to risk prognostication, no previous study, to our knowledge, has examined how these factors may interact or contribute to the underlying biology associated with aggressive prostate cancer.

Here, a methodology was developed for automated quantitative analysis of protein expression in digital microscopy images based on routinely processed immunohistochemistry (IHC) slides, and it was used to preliminarily explore a putative connection between proliferation and vascularity in the context of Gleason scores in prostate cancer. The results of the automated approach were compared with manually segmented images based on Ki-67 and CD31 protein expression. A pilot set of prostatectomy specimens (15 subjects) stained for Ki-67 and CD31 was evaluated to represent a mix of Gleason scores 6, 7, and greater than 7. Each slide was digitally scanned in high resolution format that enabled distinguishing individual cells within whole tissue sections. Three high-powered fields were chosen from each slide, and manually aligned across blocks for co-localized analysis of Ki-67 and CD31. The whole sections were then evaluated and the results compared to those obtained from individual sections. The results suggest that proliferative vascular index (PVI, defined as the ratio of Ki-67 to CD31 expression) is increased in sections representing high-grade prostate cancer. This association persists even when the automated image processing approach is applied to whole blocks representing highly heterogeneous sections of prostate cancer as commonly encountered in clinical practice. Further, an algorithm is developed to automatically extract data from Tissue Microarray (TMA) samples into rows and columns of a matrix, and test the capability of the automated approach to process TMA image samples in high throughput.

### Previous work regarding automated histology and IHC analysis

Computer aided analysis of digitally processed histologic images has been of increased interest over the past two decades [12]. The focus has been development and application of increasingly complex image analysis algorithms applied to routinely-processed hematoxylin and eosin (H&E) stained-sections to help pathological diagnosis and grading of various cancers. In particular, a variety of approaches such as Markov random field algorithms [13], color and structural morphometry [14], and graphing algorithms [15] have been proposed to improve prostate cancer assessment.

CRImage is a prime example of an automated digital pathology workflow developed to quantify epithelial, stromal, and immune cells on H&E sections [16]. This method was developed to refine results of genomic studies commonly based on analysis of an admixture of tissue elements, and has been applied to improve the resolution of breast [16] and ovarian cancer [17] studies. CRImage is capable of classifying cells, segmenting samples, and calculating tumor cellularity, in addition to native statistical analysis capabilities provided by the underlying programming language R. Although CRImage is capable of image segmentation and sample classification without requiring user input, commands must be entered for every desired process on the image, and each image must be processed individually. Further, CRImage is limited to samples stained for H&E.

Computer-aided techniques (e.g., implemented using MATLAB) have been extensively used for cell counting [18–20]. These techniques tend to have more limited application that CRImage, providing information mainly on how many cells are present and not necessarily analyzing the characteristics of these cells. The techniques are often intended for use with either dark field microscopy or grayscale images that have high contrast between the items of interest and the background, simplifying separation of cells from the background. Many of the techniques currently do not support processing DAB-stained images.

Computer-aided image quantitation has also been combined with immuno-detection technologies to study the spatial arrangement of cells and protein expression in various forms of cancer. A common application of these automated technologies has been for quantitation of Ki-67 positive nuclei in manually defined regions of interest [21, 22]. Other approaches broaden this strategy by applying machine learning algorithms to automate feature extraction of digital images annotated by a trained human expert. The Genie Pro software algorithm is an example of this approach, originally developed for analysis of satellite imagery but later applied to automate feature extraction from digital histologic images [23]. A commercial implementation of the algorithm (Genie Histology Pattern Recognition System; Leica Biosystems) has been used to quantify and correlate expression of multiple protein markers in relation to clinical outcome in prostate cancer [24, 25]. While the approach has been successful in analysis of multiple markers in tissue microarray cores, the system is not fully automated as it may require a pathologist to identify tumor regions. As Genie Pro is a stochastic learning algorithm, individual results with the same training set may vary, and the nature and weightings of features used by the algorithm may not be readily discernible to the user. There exist other approaches to quantify protein expression in tissue (such as automated quantitative analysis (AQUA) [26]), which rely on immuno-fluorescent detection that is not commonly used in clinical settings.

Accurate detection of cores in TMA samples has been previously explored using various gridding methods. Some techniques may not detect weak signal cores [27], or only work for cores regularly arranged in columns and rows [28]. For example, the technique in [29] does not account for geometric transformations and rotations often exhibited by TMA samples. Other techniques are not fully automated [29–31]. Recently, an automated technique for accurate detection and localization of tissue cores based on geometric restoration of the core shapes was presented in [32], without making assumptions about the grid geometry. The technique uses hierarchical clustering combined with the Davies-Bouldin index for cluster validation to estimate the number of cores, from which the core radius is estimated and refined using morphological granulometry.

## Methods

### Patient derived samples

A correlative science protocol for acquisition of clinical data and pathologic specimens for a prostate cancer databank was approved by the Institutional Review Board at the University of Southern California. Written informed consent was obtained from all patients prior to radical prostatectomy. Subjects were selected into 3 groups (5 subjects per group) to represent Gleason scores 6, 7, and 8 or 9. Representative tissue blocks (1–2 per subject) were selected for analysis from each subject. In total, 23 whole tissue blocks from 15 subjects were analyzed for this study. The demographics are summarized in **Table 1**.

**Table 1:** Patient demographics of the samples evaluated in this study.

				Gleason Grade		
Subject #	PSA (ng/ml)	Age (yr)	T Stage	Primary	Secondary	Score
1	3.36	50.2	pT2a	3	3	6
2	3.27	50.2	pT2c	3	3	6
3	5.30	54.9	pT2c	3	3	6
4	7.40	64.0	pT2c	3	3	6
5	11.80	69.0	pT2c	3	3	6
6	7.2	59.0	pT3a	3	4	7
7	9.13	63.7	pT3a	3	4	7
8	7.00	58.3	pT2c	3	4	7
9	18.20	64.2	pT2a	3	4	7
10	13.25	62.6	pT2c	3	4	7
11	14.9	67.7	pT3b	3	5	8
12	6	57.9	pT2c	3	5	8
13	7	65.8	pT2a	4	4	8
14	5.5	61.9	pT2c	4	5	9
15	2.4	63.3	pT3b	4	5	9

### Immunohistochemistry

Formalin-fixed paraffin-embedded 5 μm sections cut sequentially were obtained from each block, mounted on slides, and baked at 60 C for 1 hour. Slides were loaded into Leica Automated Bond III system and stained using ready to use dilutions of antibodies against Ki-67 (MIB1) or CD31 (PECAM-1) and Novocastra Bond Polymer System (Leica Biosystems). Slides were stained with these antibodies in consecutive (parallel) pairs in order to evaluate the protein expression in close spatial proximity. Specimens were de-hydrated, cover slipped with permount, and scanned using the ScanScope CS system (Aperio/Leica Biosystems) to obtain digital microscopy image files at 40X magnification (0.249 μ/pixel). Lower resolution regions of interest (ROIs) were selected from the whole block images using ImageScope (version 11.2.0.780) from Aperio Technologies for initial analysis and training. ROIs were chosen to represent predominant Gleason patterns present for each block (3 fields per slide) and exported as uncompressed, down-sampled TIFF files at 10X equivalent magnification (1652 x 992 pixels, 24 bit depth, 0.79 μ/pixel) from each whole block image.

### Automated image processing

**Fig 1:**
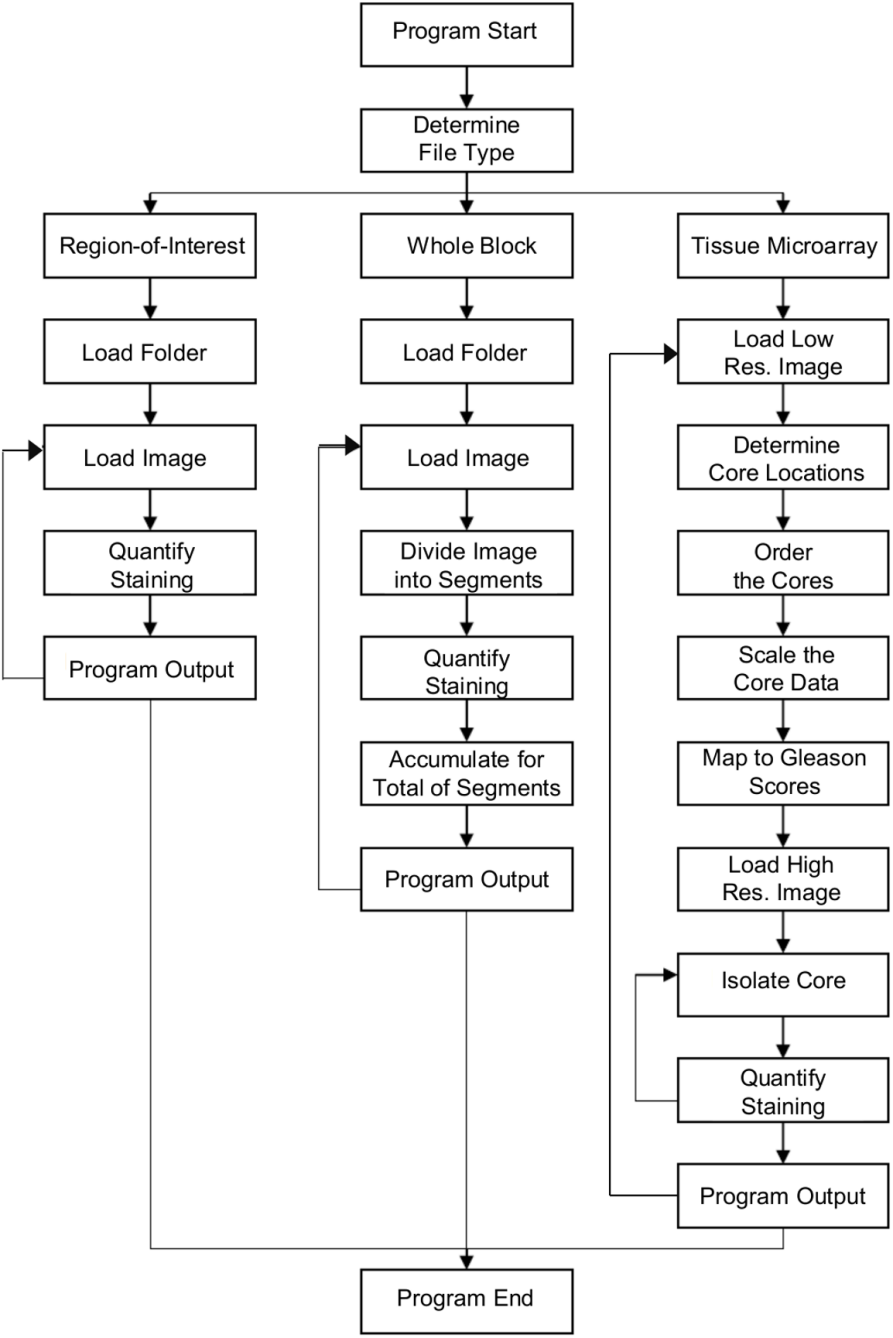
Overview of program workflow.

The overall program workflow is summarized in Fig 1. The program was implemented using Matlab version 8.3 (R2014a) on a laptop computer running Windows 8.1 (64 bit). The laptop had an Intel 1.80 GHz Core i7-4500u CPU and 8 gigabytes of physical memory.

### Determination of sample vs. background pixels

The images consist of background tissue that appears various shades of blue and stained tissue that has a reddish-brown appearance. To determine where the sample is in an image, the program determines where the background is not present. The program evaluates each pixel's RGB color values to make this decision, e.g., a perfect white would have 255 for all three RGB values, while a perfect black would have 0 for all three. The image backgrounds are not perfectly white, but have values typically >230. The samples have lower pixel values for red and/or blue based on whether or not the associated pixels have taken up the stain, since samples will usually be darker in that location. The program examines each RGB value stored for each pixel and evaluates whether the value is relatively high and whether the values are consistent for R, G, and B. If these conditions hold then the pixel is determined to be background; otherwise, it is part of the sample.

### Determination of positive vs. negative stain

Determination of positive stain is done with pixels identified to belong to the sample. Both the stained and unstained areas can vary drastically in terms of darkness (darker being associated with lower RGB values). Thus, one cannot, for example, simply compare B values for two different pixels to determine which one is "blue-er" because a darker stained area may actually have a lower blue value than a lighter non-stained area (e.g., Pixel 1 has (R,G,B) = (175,175,125), yielding blue as dominant color, while Pixel 2 has (R,G,B) = (75,175,100), yielding red as dominant color). To determine if a pixel is part of a stained area of the sample, the program evaluates the RGB values relative to each other. All parts of the sample typically have a considerable amount of blue present, so the criterion is how the red and green values compare to the blue value. If red and green are relatively higher than blue (i.e., blue is the dominant color), then the pixel is considered "unstained," and if they are lower (particularly the red value), then the pixel is considered "stained."’

Specifically, the program checks each R, G, and B value to ensure that at least one of them is below 125, and then it compares the B value to αR and to βG, were (α, β) was set to (1.00,1.25). If blue is the dominant color, then the pixel is determined to be normal tissue; otherwise, it is considered to be stained.

### Processing of region of interest images

The program allows for the continuous processing of multiple images provided that they are all located within the same folder. The program collects size information about the image to create a blank composite image of duplicate height and width. The first step is to analyze the image to separate background from the sample. As the program runs through the ROI pixel by pixel, it sets the corresponding pixel in the composite image to either black if it is background or white if it is sample. The sum total of all the white pixels is taken to determine the area occupied by the sample. The program runs over the original image again to separate pixels with stain from the rest of the image. If the program determines a pixel to have stain, it will leave the corresponding location in the composite image white, while turning the location black if without stain. The total number of white pixels in the composite image is then calculated to find the area occupied by the stain. To evaluate the accuracy of the program, regions of interest were manually evaluated using Gimp software to extract the percent of tissue stained, and the results were compared to those obtained with the program.

### Processing of whole block images

The processing begins by saving the whole block images in the.SVS file format. In order for the program to process a series of images, the user must set up a folder including all of the desired images. The program reads that image and collects its height and width. Due to potential memory limitations associated with the size of the images, the program segments each image of the whole tissue block using a four by four grid, creating sixteen segments equal in size. The program then creates a separate image of size equal to that of the sixteen segments to be used during the analysis.

The program first analyzes each pixel within a segment by categorizing it to either be sample or background. The corresponding pixel location in the composite image is then changed to black or white, respectively. Next, each pixel of the segment is categorized to either contain brown stain or not in a method similar to the sample/background categorization. The number of white pixels present in the composite image is then counted to be representative of the amount of stained sample in this segment. The amount of non-stained sample is calculated by subtracting the amount of stained sample from the amount of total sample for the segment. This process continues for each of the remaining segments, adding the values found for each segment to the cumulative totals being tracked.

### Processing of tissue microarray (TMA) images

**Fig 2:**
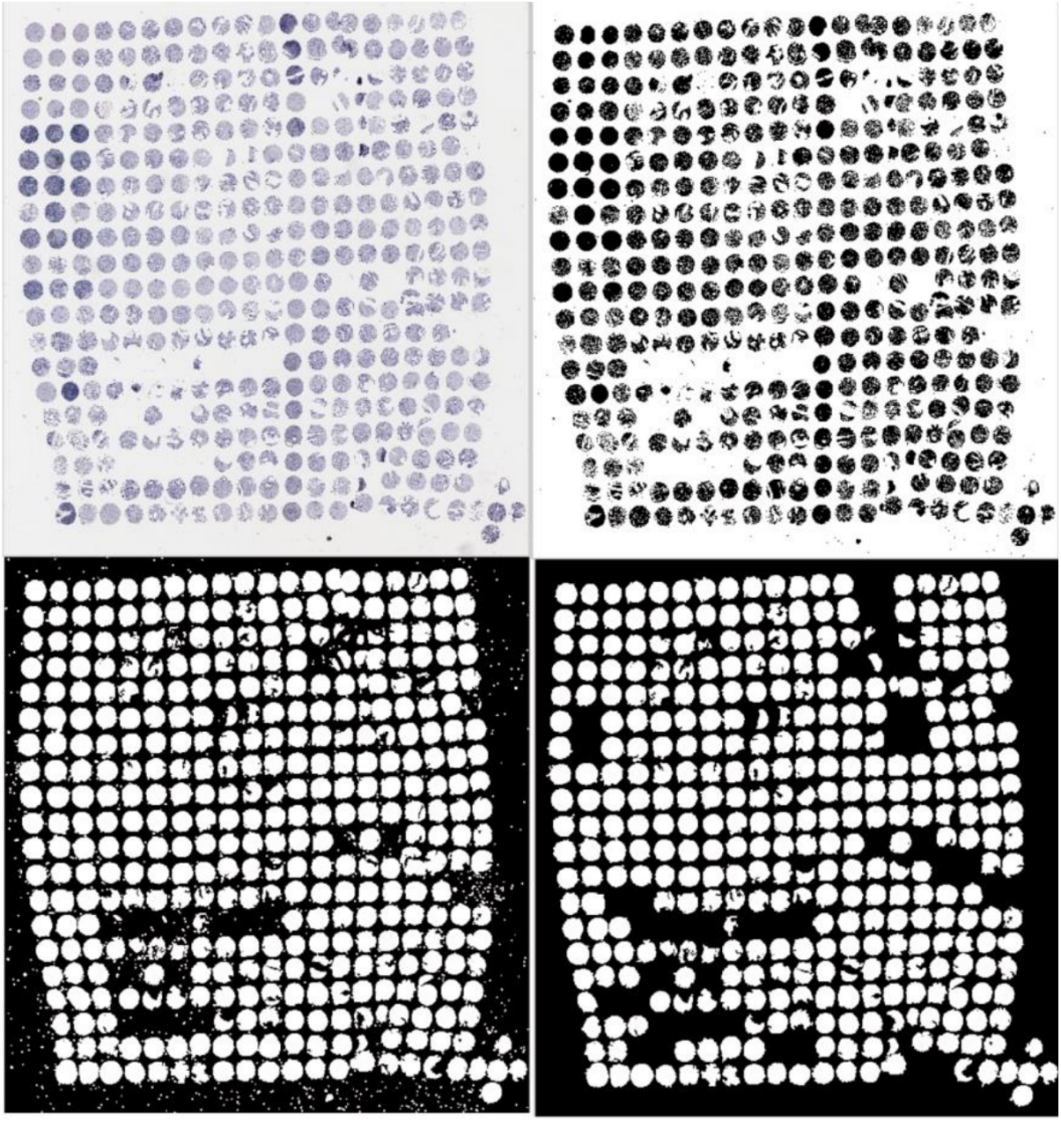
Example of processing of TMA images to identify core samples.

A scaled down version of the TMA image is first loaded into the program to extract necessary information while conserving system memory. Information regarding the size of this image is gathered in order to create an all-black image of the same size. The first step of segmenting the cores on the TMA’s is to remove the background. If a pixel is determined to be part of the background, then the corresponding pixel on the black image is changed to white. Some cores can be fragmented; therefore, it is necessary to manipulate the image to merge those sections. This is accomplished by creating a structuring element, using it to erode/dilate the image, creating a perimeter around the outside of the object, and filling all of the holes within the object to leave one whole area representing the location of the core. In order to remove unwanted objects as well as cores that may have merged together, the areas of the objects are analyzed, turning all areas that are either exceedingly small or large back to all black (Fig 2).

Centroid locations of all the remaining white objects are collected and exported to a matrix with each row representing a different core, the first column representing the horizontal position of the centroid, and the second column representing the vertical position of the centroid. A third column is then created and filled with the value equal to the sum of the first two columns and the entire matrix is sorted by the third column to find the core likely nearest to the image origin, i.e., the top left corner of the image, assuming uniform placement of cores in the TMA.

A separate location matrix is created with size equal to that of the grid of cores present on the TMA. The program references the centroid location of the original core to search for adjacent cores, focusing first on completing the first column by searching the centroid matrix for entries with similar horizontal position to the previous core and vertical positions within a specified range away from the previous core. If a centroid is found to be in this expected location then that core is referenced in the location matrix by filling its relative position with the row number of its entry in the centroid matrix and the process is repeated to find the next core along the column. If no core is found for an expected area, then the search is expanded to look for a centroid that would be located two places on the grid away from the previous core, and the search is expanded in this manner until the next core is found. Once the first column is complete, the program checks that a proper amount of samples were found to create a baseline for the row information off that column. If the amount of samples is found to be insufficient, the program will instead attempt to create a baseline from the first row rather than the first column. Once the baseline row/column is established, the program will fill in the columns/rows by similar means to the previous method, using the baseline row/column as the original reference.

Two columns are added to the centroid matrix when the location matrix is complete, which are filled with the row and column information for where that sample was placed in the location matrix. This results in the assigning of a core to both a column and row for future reference. Since the TMA images used in this study were oriented opposite of the conventional numbering system being used for the rows and columns of the samples, the value of the row and column assigned to each sample is inverted so that samples assigned to the first row or column are now labeled as being in the last row or column and vice versa. The large scale TMA image is then read into the program so that the cores can be analyzed at higher resolution. Since location information gathered for the centroid locations does not correspond to their locations on the larger image, the pixel locations are scaled according to the image sizes.

The data are read from a spreadsheet consisting of the row location, column location, and Gleason scores of the cores on the TMA as graded by a pathologist. This data is used to assign the proper Gleason score to each core that is found by the program. The scaled location data from the centroid matrix is used to identify the centroid locations on the large scale image. From the area surrounding the centroid location, a composite image is generated that consists of only one sample. Another image of the same size as the single core image is created to document the analysis of the core. First, the core is analyzed pixel by pixel to determine whether each pixel is part of the background or the sample in question. If it is background, then the pixel is set to black, whereas if the pixel is sample, it is set to white. The program takes the total of all the black pixels in the analysis image to document how much of the image is background and the sum total of all the white pixels to determine how much of the image is sample. Next, the core is analyzed pixel by pixel to determine if brown stain present, in which case the corresponding pixel is set to black, otherwise the corresponding location is set to white. From here, the percent of the core that is stained is found, and the whole process is repeated on the next core until all cores have been analyzed. Finally, the centroid matrix, consisting of core centroid locations, row and column of the core, Gleason score, and the percentage that had brown stain, are exported to an excel file.

### Statistical analysis

Analyses used the two-tailed Student’s t-test with a significance level of 0.05. Statistically significant results are illustrated with an asterisk (*) in the Results.

## Results

**Fig 3:**
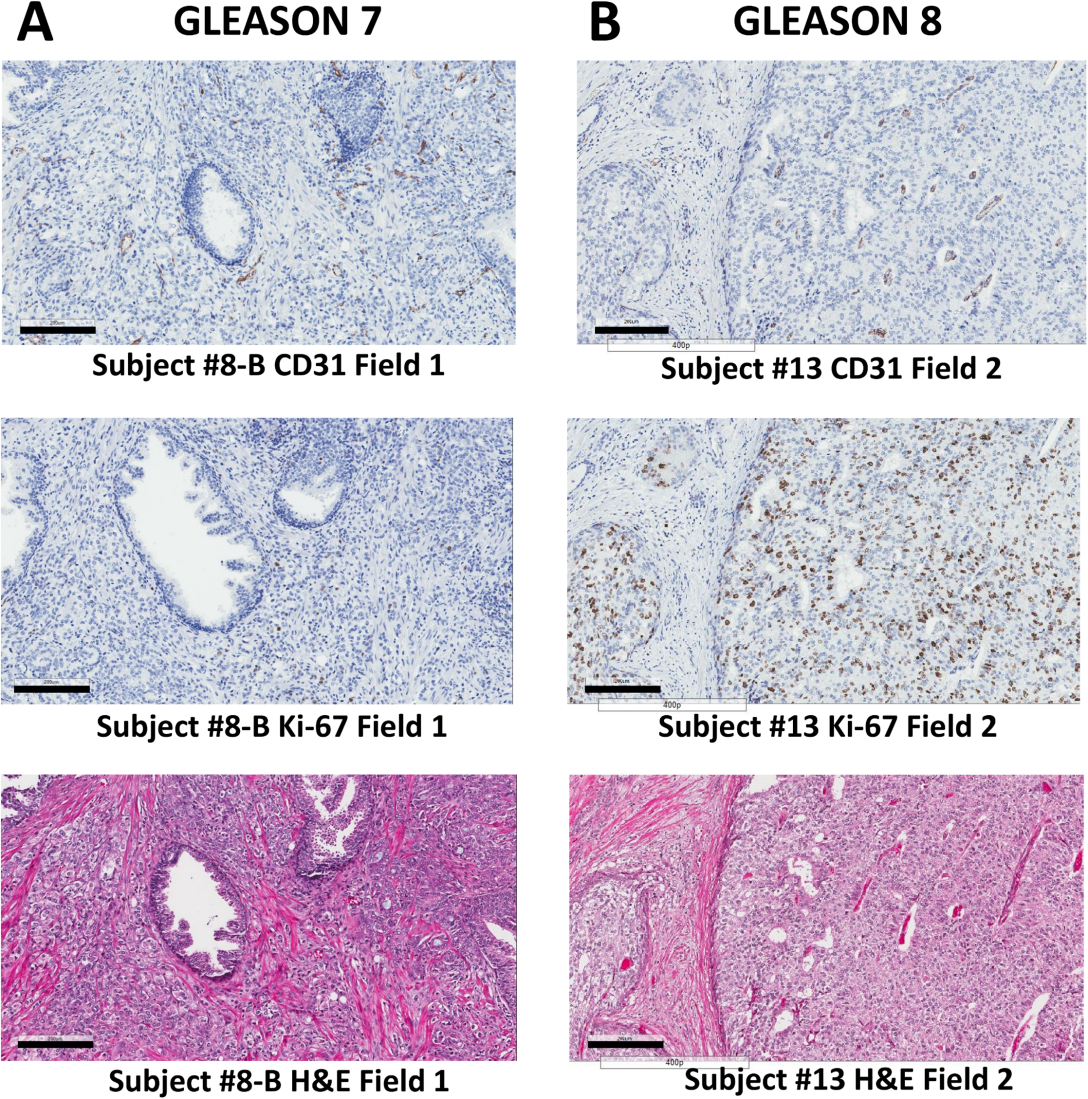
**Representative ROI histology images for (A Column) low grade (Gleason score 7) and (B Column) high grade (Gleason score 8) prostate cancer.** Staining is shown for CD31 (vascularization), Ki-67 (cell proliferation), and H&E (cell viability) for consecutive slides. Bar, 200 μm.

**Fig 4:**
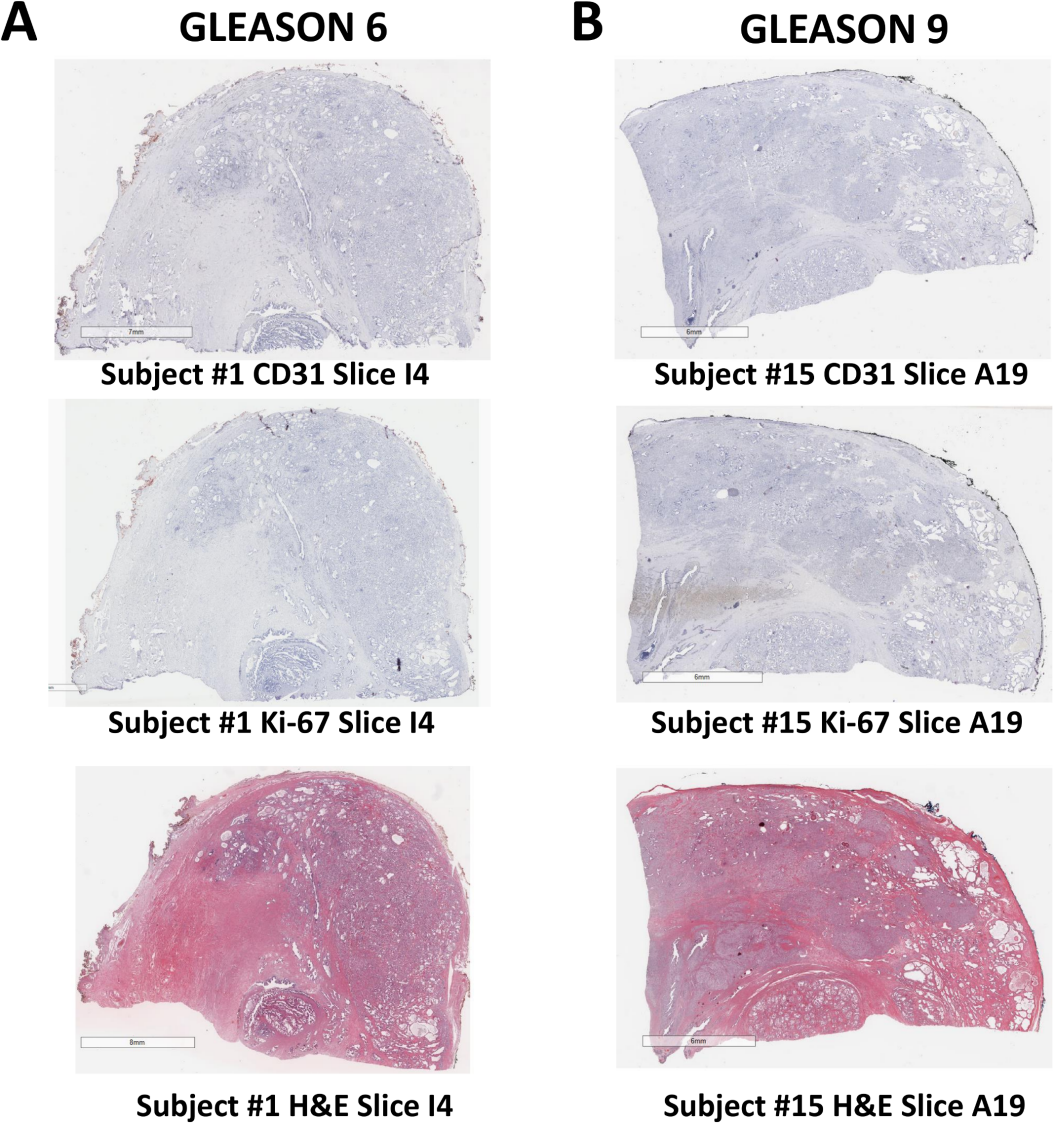
**Representative whole tissue block images for (A Column) low grade (Gleason score 6) and (B Column) high grade (Gleason score 9) prostate cancer.** Staining is shown for CD31 (vascularization), Ki-67 (cell proliferation), and H&E (cell morphology) for consecutive slides. Bar, 6 mm.

Representative ROI histology images for low grade and high grade prostate cancer are shown in Fig 3. Staining is shown for CD31 (vascularization), Ki-67 (cell proliferation), and H&E (cell morphology) for consecutive slides. Representative whole tissue block images for low grade and high grade prostate cancer with the same staining types and consecutive to each other are presented in Fig 4.

### Comparison of manual to automated program results

**Fig 5:**
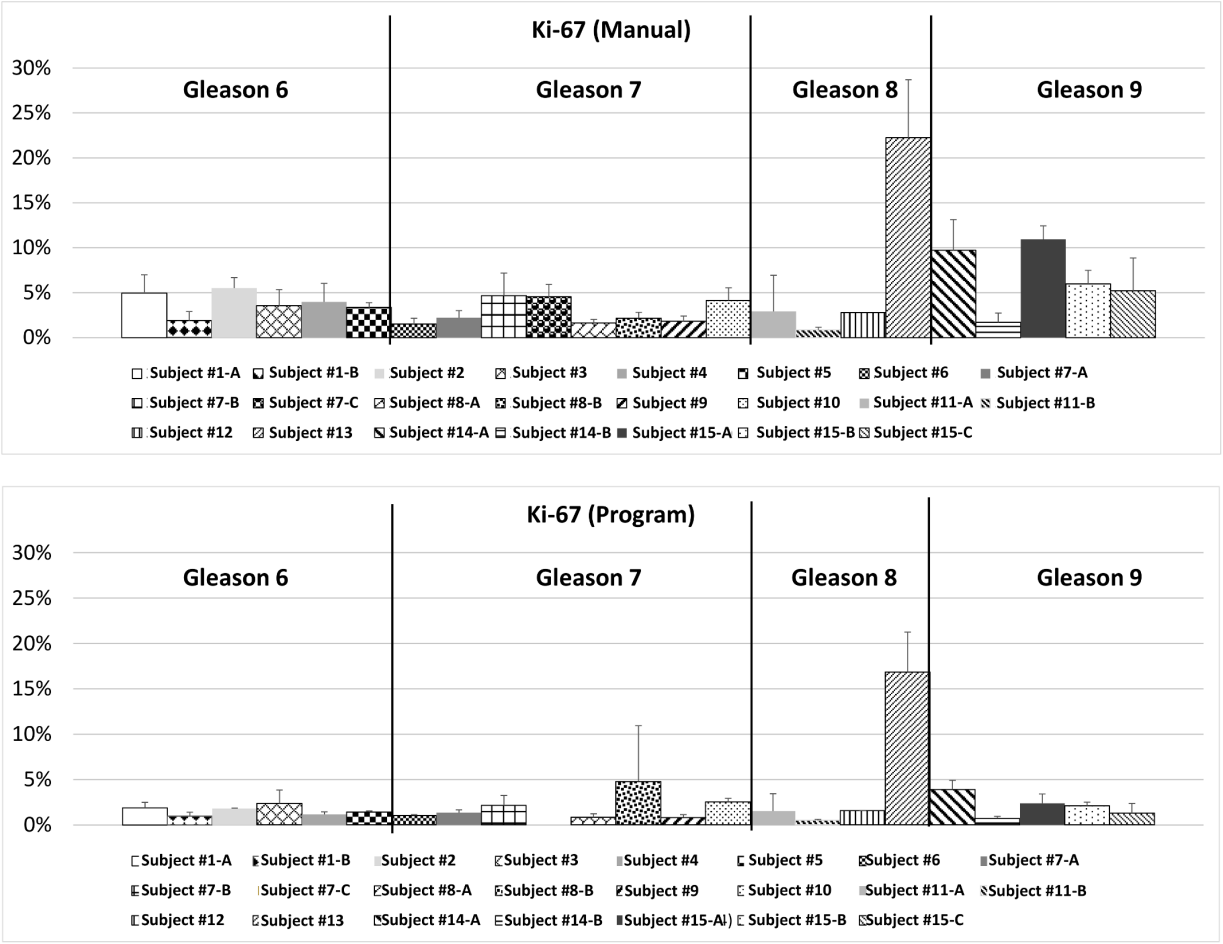
Comparison of results of the ROI analysis between the manual method and the program in terms of Ki-67 stain.

**Fig 6:**
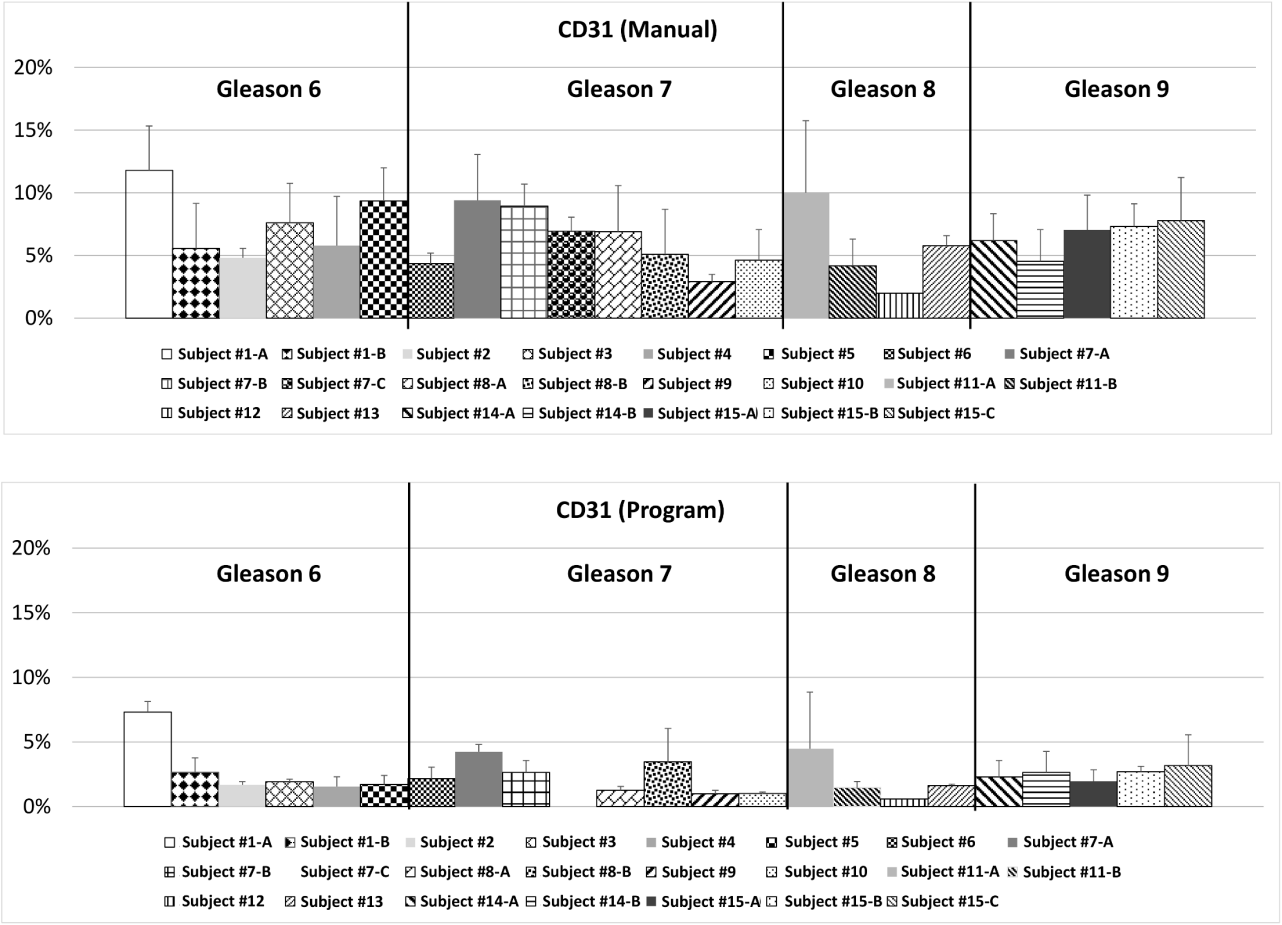
Comparison of results of the ROI analysis between the manual method and the program in terms of CD31 stain.

**Fig 7:**
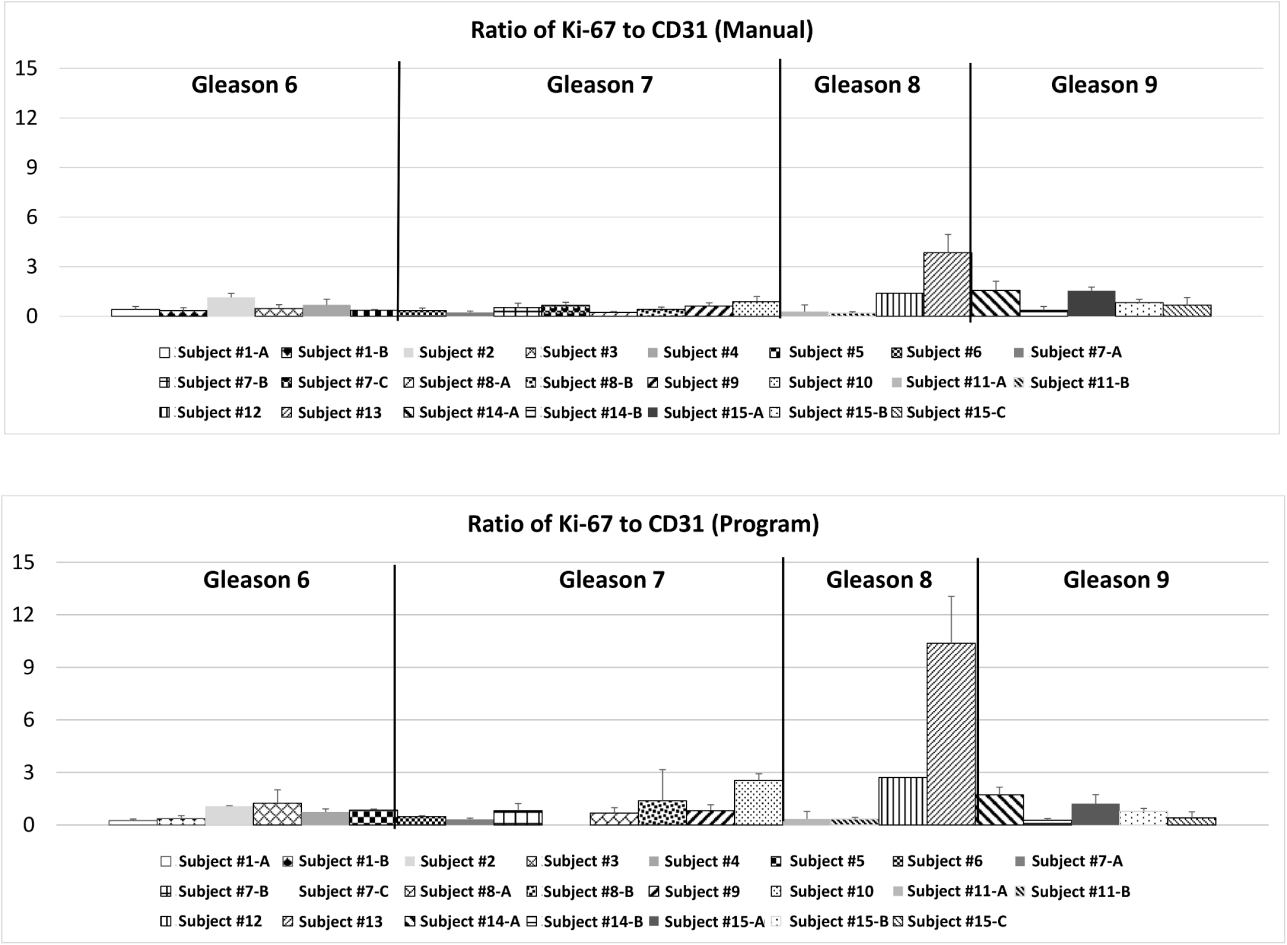
Comparison of results of the ROI analysis between the manual method and the program in terms of Ki-67 to CD31 stain ratio.

The process of creating an automated program began with designing a system that could replicate results from the manual analysis. The manual evaluation was initially used to analyze various ROIs from existing tissue slices, and these were chosen as a benchmark. The program ran analysis for these regions having both Ki-67 and CD31 stains, and calculated the ratio of the two for each region. For both stains the program underreported in comparison to the manual method but followed a similar proportional difference between samples (Figs 5 and 6). This resulted in the ratio of the Ki-67 stain to the CD31 stain being consistent with the data collected manually (Fig 7). Each figure shows the results in two separate panels to allow for consistent comparison of each method between patients.

**Fig 8:**
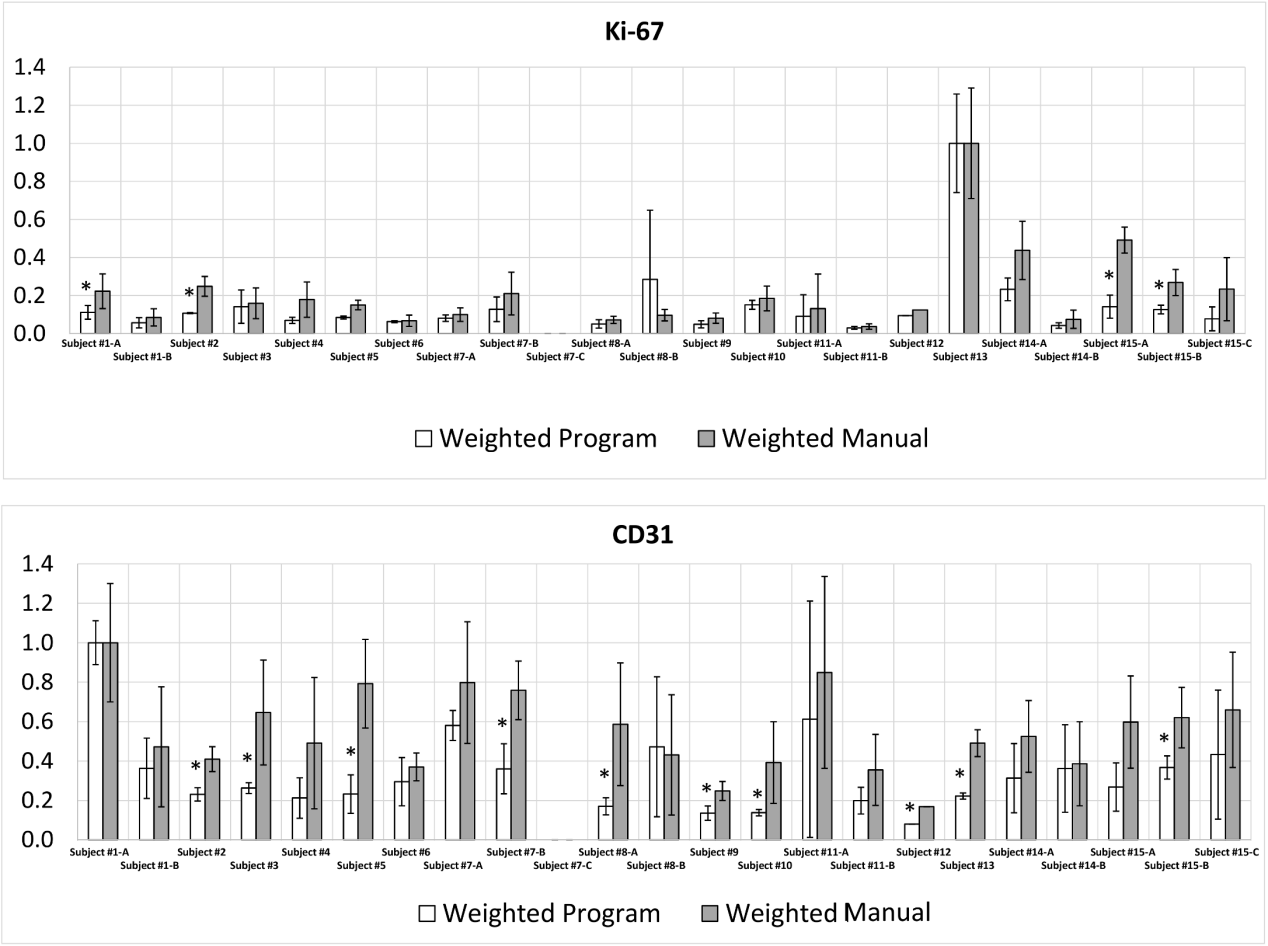
Comparison between normalized manual and automated results of the ROI analysis for (a) Ki-67 stain and (b) CD31stain data sets.

In order to further evaluate whether the program could obtain results similar to the manual process, the data were normalized with respect to its method. The reported average stain concentrations and the corresponding standard deviations for each sample were normalized by the sample reported to have the highest stain concentration. The resulting data were plotted to visualize the comparison (Fig 8). The results indicate that the program mostly reported similar relative results as the manual method, showing that 30 out of 44 data points (68%) were not significantly different between the two methods.

### Analysis of epithelial and stromal protein expression

**Fig 9:**
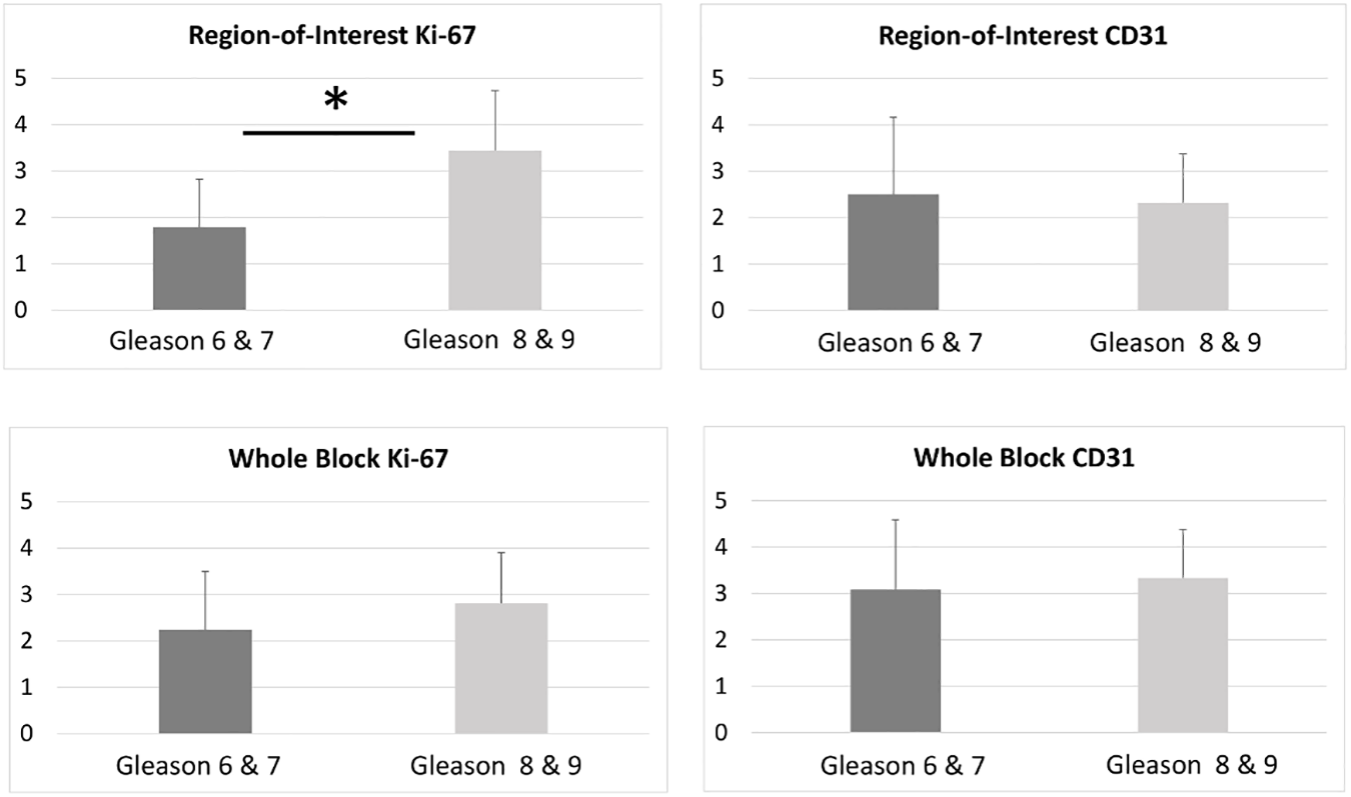
Epithelial (Ki-67) and stromal (CD31) protein expression in ROI and whole tissue blocks. Asterisk signifies statistical significance between the two Gleason score categories.

The program was applied to automatically analyze the region-of-interest samples as well as the whole block specimens. For each sample within the data set there was an image possessing Ki-67 (proliferative) stain and a separate image possessing CD31 (vascularity) stain for consecutive regions within the tissue. Once every sample had been analyzed, the samples were separated into low (6 or 7) and high (8 or 9) Gleason categories. The results showed that the difference between the categories in the presence of the Ki-67 stain was significant for the ROI samples but not for the CD31 stain (Fig 9).

### Analysis of ratio of epithelial to stromal protein expression

**Fig 10:**
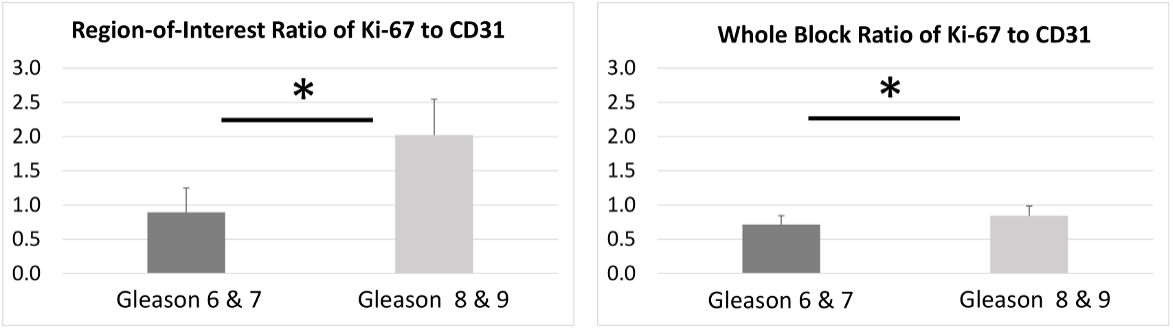
Ratio of epithelial (Ki-67) to stromal (CD31) protein expression in ROI and whole tissue blocks. Asterisk signifies statistical significance between the two Gleason score categories.

The ratio of Ki-67 (proliferative) stain to CD31 (vascularity) stain observed by the program in the ROI samples as well as the whole block specimens was calculated. The results showed that there was a statistically significant difference for the Ki-67/CD31 ratio in both image categories (Fig 10), with the ratio being higher for the more advanced disease (Gleason 8 and 9).

### Evaluation of capability to analyze TMA images

**Fig 11:**
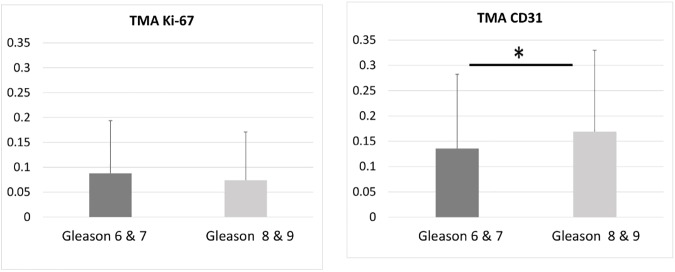
Epithelial (Ki-67) and stromal (CD31) protein expression, and the ratio between these two values in TMAs. Asterisk signifies statistical significance between the two Gleason score categories. Note that the TMA samples were not used to determine the Gleason categories.

The program’s ability to process TMA images was evaluated in high throughput, being aware that these samples were not used for the patient cancer staging and thus the results were not comparable to the ROI or whole slide data analysis. The difference in Ki-67 stain between the two categories was not found to be significant but the difference in CD31 stain was significant (Fig 11).

## Discussion

A framework was developed for automated analysis of protein expression in high-resolution digital microscopy images of whole tissue blocks processed with routine IHC reagents. A variety of practical and technical factors have limited the adoption of tissue based protein expression biomarkers of most prior research studies for routine clinical practice. First, immune-detection techniques are notoriously difficult to standardize across samples and laboratories. Thus, it is difficult to quantitatively score any IHC marker for clinical diagnostics. Further, intra-sample variability is a major issue in prostate cancer as even the Gleason grade can vary in different parts of the sample surgical specimen. Many biomarker studies are based on observations made using tissue microarrays (TMAs). While TMAs have inherent advantages in minimizing variation in staining techniques and may offer lower costs, the results are still subject to sampling effects as only a handful of small cores are usually obtained from each tumor block/subject. Thus, findings based on TMAs may fail to validate when tested on whole tissue blocks. Previous work has indicated that it is unclear to what extent TMAs are able to represent whole sections, and whether TMA data can be reliably used for clinico-pathological correlations and survival analysis [33].

The technique presented here was applied to explore protein expression patterns in relation to Gleason grade in prostate cancer. It has been suggested that molecular factors play a critical role in Gleason grade. In one seminal study, a transcriptional microarray was used to compare expression of genes in Gleason 3 versus Gleason 4/5 tissue areas separated by laser capture micro-dissection [34]. A significant correlation was observed between high levels of monoamine oxidase A (MAOA), defender against death (DAD1) protein, and HSD17β4 with Gleason grade. Other studies have shown expression of epithelial differentiation markers such as androgen receptor and prostate specific membrane antigen [35, 36], hypoxia (carbonic anhydrase IX, hypoxia inducible factor 1α) [37], and PTEN pathway activation [38] are increased in higher grade cancers. More recently, several molecular diagnostic or “genomic” assays have been developed to supplement standard clinical and pathologic risk stratification based on expression analysis [39]. Genes for analysis in this assay are chosen such that they do not directly correlate with Gleason grade so as to provide independent prognostic information. Furthermore, the targets in these assays are generally chosen based on technical factors such as robust expression signatures across samples and statistical validity in multi-parametric models, rather than because they impart independent biologic information concerning the cancer in a specific patient.

This study chose to compare expression of proliferation and vascularization as biomarkers particularly relevant to tumor grade in prostate cancer. Both proliferative index and vascularity have been associated with poor outcomes in prostate cancer [9–11]. Although the number of patient samples is small, the results suggest a biologic basis for how tumor and host factors interact in relation to histologic grade. Proliferation is shown to be less dependent on vascularity in higher grade prostate cancers. Thus, unregulated proliferation may be a key feature which distinguishes high-grade from low-grade prostate cancer and we posit that constitutive up-regulation of proliferative signals may be a key factor underlying the more aggressive clinical course of higher-grade prostate cancers, which will be evaluated with larger populations in future studies.

Overall, there is an opportunity to develop improved techniques to study spatial arrangement of protein expression in relation to Gleason grade in prostate cancer, and the goal of this work is to offer a first step in this direction. We recognize that a limitation of the study is that the analyses are based on a limited set of samples, and that the similarity between manual and program measurements could be higher. We plan to expand the analysis to a larger dataset in the future, for which updates to the techniques will be included to increase accuracy.

In conclusion, an approach was developed for automated analysis of co-localized protein expression in histologic sections of prostate cancer, and initially evaluated it with three types of patient histology information. The underlying biological hypothesis is that the Gleason grade is determined by the interaction between cancer and host factors. Examples of “host factors” include stromal reaction, immune status, and tissue stiffness. The analysis based on region-of-interest and whole block slides preliminarily suggests that proliferation (Ki-67 expression) is relatively independent of vascularity (CD31 expression) in high-grade prostate cancer. A higher number of cases than the 15 patients here will need to be evaluated in order to support this analysis. Longer term, we aim to further develop an integrated experimental/computational approach to model the epithelial and stromal compartments in prostate cancer, as we have previously explored for lymph, brain, pancreatic, breast and cervical cancers [40–48]. Such a system would enable characterization and modeling of the prostate tissue from the cellular- to the organ-scale for the identification of biological characteristics that determine histologic grade and that may ultimately drive patient prognosis.
